# Breast-conserving surgery without axillary surgery and radiation versus mastectomy plus axillary dissection in elderly breast cancer patients: A retrospective study

**DOI:** 10.3389/fonc.2023.1126104

**Published:** 2023-03-20

**Authors:** Ying Zhong, Zhe Wang, Yali Xu, Yidong Zhou, Feng Mao, Songjie Shen, Qiang Sun

**Affiliations:** Department of Breast Disease, Peking Union Medical College Hospital, Beijing, China

**Keywords:** breast cancer, elderly patients, breast-conserving surgery, without axillary surgery and without radiotherapy, triple negative breast cancer

## Abstract

**Background:**

The high relative mortality rate in elderly breast cancer patients is most likely the result of comorbidities rather than the tumor load. Foregoing axillary lymph node dissection or omitting radiotherapy after breast-conserving surgery (BCS) does not affect the prognosis of elderly breast cancer patients. We sought to assess the safety of breast-conserving surgery without axillary lymph node dissection as well as breast and axillary radiotherapy (BCSNR) in elderly patients with early-stage breast cancer.

**Methods:**

We retrospectively included 541 consecutive breast cancer patients aged over 70 years with clinically negative axillary lymph nodes in one clinical center. Of these patients, 181 underwent mastectomy plus axillary lymph node dissection (MALND) with negative axillary cleaning and 360 underwent BCSNR.

**Results:**

After a median follow-up of 5 years, there was no significant difference between the BCSNR and MALND groups in either distant recurrence-free survival (DRFS) (*p*=0.990) or breast cancer-specific survival (*p*=0.076). Ipsilateral axillary disease was found in 11 (3.1%) patients in the BCSNR group and 3 (1.7%) patients in the MALND group; this difference was not significant (*p*=0.334). We did not observe a significant difference in distant recurrence between the groups (*p*=0.574), with 25 (6.9%) patients in the BCSNR group experiencing distant recurrence compared to 15 (8.3%) patients in the MALND group. Our findings did show a significant difference in ipsilateral breast cancer recurrence (IBTR), with 31 (8.6%) patients in the BCSNR group experiencing IBTR compared to only 2 (1.1%) patients in the MALND group (*p*=0.003).

**Conclusion:**

BCSNR is a safe treatment option for elderly breast cancer patients with clinically negative axillary lymph nodes.

## Introduction

Breast cancer is the most common type of malignancy among women, with an incidence rate of 29% (1). Breast cancer has the highest mortality rate among all malignant tumors in women aged 20–59 years and has the second highest mortality rate in patients over 60 years of age (2). Patients over 65 years of age account for 40% of all breast cancer patients while those over 75 years of age account for 20% of all cases. It has been reported that both the 5-year and 10-year relative survival rates of patients aged 70 years or older are lower than those of patients aged 40–70 years (3). However, in elderly patients, breast cancer-related mortality is lower than that in other age groups (4). Thus, breast cancer is not the main factor affecting the survival of elderly patients (5). Instead, the high relative mortality rate in elderly breast cancer patients is most likely the result of comorbidities, poor physical condition, or other parameters associated with age (6).

Older women are more likely to have estrogen receptor-positive (ER+) and progesterone receptor-positive (PR+) breast cancer than younger women (7). The rate of hormone receptor-positive (HR+) cancer increases from more than 60% in women aged 30–34 years to 85% in women aged 80–84 years (8). Breast cancer is classified into four molecular types based on the immunohistochemical (IHC) detection of ER, PR, HER2, and Ki-67: luminal A, luminal B, HER2-positive, and triple-negative (9). Previous studies have shown that with advancing age, the rate of luminal patients increases and prognosis improves (10). As a result, breast cancer-related mortality decreases with age (4).

Foregoing axillary lymph node dissection does not affect the disease-free survival or overall survival (OS) of elderly breast cancer patients with clinically negative axillary lymph nodes (11). Similarly, omitting radiotherapy after breast-conserving surgery (BCS) does not affect the survival of elderly breast cancer patients (12). For some patients, the radiotherapy-related damage to the heart and lungs may exceed the benefits of radiotherapy, especially in those with a long history of smoking (13–15). Given that distant metastasis from breast cancer depends on the biological characteristics of the tumor itself, axillary surgery is a staging tool rather than a means of reducing mortality (16). According to our knowledge, there is no study on the safety of BCS foregoing axillary lymph node dissection and without breast and axillary radiotherapy in elderly patients. We therefore sought to determine whether foregoing axillary lymph node cleaning as well as breast and axillary radiotherapy after BCS negatively affects the survival of elderly breast cancer patients with clinically negative axillary lymph nodes. We retrospectively analyzed a group of breast cancer patients over 70 years of age who had clinically negative axillary lymph nodes to determine whether the patients’ survival and rate of recurrence differed according to treatment of breast-conserving surgery without axillary lymph node surgery as well as breast and axillary radiotherapy (BCSNR) versus mastectomy plus axillary lymph node dissection (MALND).

## Materials and methods

### Patient data

The data of breast cancer patients who received treatment at our hospital have been conserved in a database since 1975, including the data on age, surgical method, radiotherapy, chemotherapy, endocrine therapy, concomitant diseases, recurrence, and metastasis. Hard copies of patient records were scanned and filed electronically, and the data relevant to this study were extracted and compiled in a new database. Since 1975, more than 2000 breast cancer patients over the age of 70 have undergone surgery and other treatments at our hospital, of which 970 were included in this study from 2000 to 2015. Of these from 2000 to 2015, 360 patients had clinically negative axillary lymph nodes and underwent BCSNR. An additional 181 patients who also had clinically negative axillary lymph nodes underwent MALND, and their postoperative axillary lymph node status was negative (patient’s management flow chart in **Supplementary Figure 1**). All surgical procedures as well as clinical visits and other treatments occurred at our hospital. All patients provided written informed consent.

All patients were informed prior to surgery of their surgical options including BCSNR and MALND, together with the risks, advantages, and disadvantages of each option. The surgical method was chosen according to the patients’ preferences and doctors’ opinions.

All patients underwent a preoperative clinical evaluation including physical examination (PE), ultrasonography, and mammography. Clinically negative axillary lymph node status was defined as the absence of enlarged lymph nodes on PE and the absence of suspicious lymph nodes on ultrasound and mammography (no abnormal blood flow, volume increase, morphologic abnormality, or suspicious calcification). Patients with no suspicious calcification on the mammograph and no other suspicious tumors in multiple quadrants, based on ultrasound findings, underwent BCSNR. All patients with HR-positive tumors received endocrine treatment. No patients were treated with radiotherapy; however, some received a chemotherapy regimen that included capecitabine.

### Tumor characteristics

Pathological information for all patients was obtained from the Department of Pathology at our hospital. ER- and PR-positive breast cancer was defined based on positive IHC staining of > 1% of cells. HER2-positive breast cancer was defined as a breast cancer with an IHC HER2 protein positivity score of 3+. In cases with an IHC HER2 protein positivity score of 2+, fluorescence immunofluorescence hybridization was performed for HER2, with positivity defined as an average HER2 gene copy number of ≥ 6 signals/cell or a HER2/CEP17 ratio of ≥ 2 (17). T stage was categorized as follows: T1 (< 5 cm), T2 (> 2 ≤ 5 cm), T3 (> 5 cm), and T4 (any size with direct extension to the chest wall or skin). Axillary lymph node stage was categorized as follows: N0 (0 positive axillary lymph nodes), N1 (metastasis to 1–3 axillary lymph nodes), N2 (metastasis to 4–9 axillary lymph nodes), and N3 (metastasis to ≥ 10 axillary lymph nodes). Histological type was determined according to World Health Organization classification (18). In IHC, Ki67 expression was classified as low or high with a cut-off value of 14%.

### Follow-up and outcome variables

Re-examination was performed in the outpatient department every 6 months, including chest X-ray and ultrasound of the breast, upper abdomen, pelvis, and neck lymph nodes; a bone scan was performed once a year. Data on disease status or cause of death were obtained by reviewing medical records or by contacting the patient *via* phone.

For the purposes of this study, local recurrence was defined as local breast recurrence, chest wall recurrence, or ipsilateral axillary lymph node recurrence. Distant recurrence included metastases to the bone, liver, lung, and brain as well as distant skin metastases.

Distant recurrence-free survival (DRFS) was defined as survival without distant recurrence. Breast cancer-specific death was defined as death caused by breast cancer recurrence or metastasis. Non-breast cancer-specific death was defined as death caused by other concomitant diseases. Breast cancer-specific survival was defined as survival without breast cancer-related death.

### Statistical analysis

All statistical analyses were performed using R software (version 3.6.2; https://cran.r-project.org) and Statistical Package for Social Sciences (SPSS) 23.0 software. All statistical tests were two-sided, and significance was defined as a p value < 0.05. For all patients, the surgical date was taken as the starting point of analyses, and the final follow-up date was February 17, 2020. DRFS was calculated using Kaplan–Meier analysis. The associations between surgical method and local recurrence, distant recurrence, ipsilateral breast cancer recurrence (IBTR), breast cancer-specific death, and non-breast cancer-specific death were evaluated using the chi-square and Mann–Whitney U-tests. Tests for interactions between distant recurrence or breast cancer-specific death and surgery method, age, T stage, histology type, and molecular type were performed using univariate Cox regression analysis and a multivariate Cox regression model.

## Results

### Patient characteristics

We selected 541 patients with clinically negative axillary lymph nodes. The median age was 75.7 (range: 70–93) years. Of these patients, 264 (48.8%) were 70–74 years old, 153 (28.3%) were 75–79 years old, and 124 (22.9%) were >80 years old. In all the patients, 360 patients underwent BCSNR and 181 patients underwent MALND with negative postoperative axillary lymph node. In all the patients, 87 (16.1%) patients received chemotherapy with capecitabine. The majority of patients (430; 79.5%) had HR-positive tumors and were administered aromatase inhibitors. The breast cancer subtypes were distributed as follows: 430 (79.5%) patients, luminal subtype(s) (including luminal A and luminal B sub-type); 28 (5.2%), HER2-enriched subtype; and 69 (12.8%), triple-negative breast cancer (TNBC). The majority of patients had T1 and T2 stage tumors: 310 (57.3%) patients, T1 and 154 (28.5%) patients, T2. Regarding the histological type, 393 (72.6%) patients had invasive ductal carcinomas (IDC), 51 (9.4%) had *in situ* carcinomas (ISC) (including ductal carcinoma *in situ* or Paget disease), 30 (5.5%) had invasive lobular carcinomas, and 67 (12.4%) had other invasive carcinomas such as mucinous, cribriform, and medullary cancers (**Table 1**).

**Table 1:** Clinical characteristics according to operation method.

	Total		BCSNR		MALND	
	No	%	No	%	No	%
Age group						
70-74	264	48.4%	126	35.0%	138	76.2%
75-79	153	28.3%	119	33.1%	34	18.8%
80-84	94	17.4%	86	23.9%	8	4.4%
85-89	25	4.6%	24	6.7%	1	0.6%
>89	5	0.9%	5	1.4%	0	0.0%
T stage						
0	55	10.2%	41	11.4%	14	7.7%
1	310	57.3%	207	57.5%	103	56.9%
2	154	28.5%	101	28.1%	53	29.3%
3	13	2.4%	5	1.4%	8	4.4%
Uk	9	1.7%	6	1.7%	3	1.7%
Histologic type						
ISC	51	9.4%	39	10.8%	12	6.6%
IDC	393	72.6%	246	68.3%	147	81.2%
ILC	30	5.5%	24	6.7%	6	3.3%
Other invasive carcinoma	67	12.4%	51	14.2%	16	8.8%
HR						
negative	107	19.8%	61	16.9%	46	25.4%
positive	429	79.3%	296	82.2%	133	73.5%
Uk	5	0.9%	3	0.8%	2	1.1%
HER2						
negative	415	76.7%	282	78.3%	133	73.5%
positive	47	8.7%	26	7.2%	21	11.6%
Uk	79	14.6	52	14.4%	27	14.9%
Ki-67						
low	250	46.2%	187	51.9%	63	34.8%
high	229	42.3%	153	42.5%	76	42.0%
Uk	62	11.5%	20	5.6%	42	23.2%
Molecular type						
Luminal type	429	79.3%	297	82.5%	133	73.5%
HER2 type	28	5.2%	14	3.9%	14	7.7%
TNBC	70	12.9%	41	11.4%	28	15.5%
Uk	14	2.6%	8	2.2%	6	3.3%

BCSNR, breast conserving surgery without axillary lymph node surgery and radiotherapy for breast and axillary.

MALND, mastectomy plus axillary lymph node dissection.

ISC, in situ carcinomas including ductal carcinoma in situ or Paget disease.

IDC, invasive ductal carcinoma.

ILC, invasive lobular carcinoma.

Uk, unknown.

The median follow-up time of the patients was 61 months (interquartile range [IQR]= 34–88 months). Median follow-up duration was 58 months (IQR= 30–58 months) in the BCSNR group and 76 months (IQR= 41–76 months) in the MALND group.

### Effect of the surgical method on recurrence

**Table 2** shows the recurrence events including ipsilateral axillary disease, contralateral breast cancer, distant recurrence, local recurrence, and IBTR. Ipsilateral axillary disease occurred in 11 (3.1%) patients in the BCSNR group and 3 (1.7%) patients in the MALND group; the difference between the two groups was not significant (*p*=0.404). In the BCSNR group, there were 25 (6.9%) patients with distant recurrence, which was similar to the number (15 [8.3%] patients) in the MALND group (*p*=0.574). Only two patients (one in each group) presented with contralateral breast cancer during follow-up. Thirty-seven (10.3%) patients in the BCSNR group had local recurrence, which was significantly higher than that in the MALND group, where only 4 (2.2%) patients experienced local recurrence (*p*=0.001). The rate of IBTR was significantly higher in the BCSNR group (31; 8.6%) than in the MALND group (2; 1.1%) (*p*=0.003). In the BCSNR group, IBTR occurred in 24.4%, 6.1%, and 21.4% of patients with TNBC, luminal type breast cancer, and HER2-enriched breast cancer, respectively, while their counterparts in the MALND group experienced IBTR rates of 0.0%, 0.8%, and 7.1%, respectively. The differences in IBTR rates between the counterparts in the two groups were significant for patients with TNBC (*p*=0.004) and luminal type breast cancer (*p*=0.010) but not for those with HER2-enriched breast cancer (*p*=0.596).

**Table 2:** Distant recurrence, Local recurrence and IBTR according to operation method.

	Total		BCSNR		MALND		*P* value
	No	%	No	%	No	%	
Total patients							
Ipsilateral axillary disease	14	2.6%	11	3.1	3	1.7%	0.404
Contralateral breast cancer	2	0.4%	1	0.3	1	0.6%	1.00
Distant recurrence	40	7.4%	25	6.9	15	8.3%	0.574
Local recurrence	41	7.6%	37	10.3	4	2.2%	0.001
IBTR	33	6.1%	31	8.6	2	1.1%	0.003
Luminal patients							
Ipsilateral axillary disease	8	1.9%	6	2.0	2	1.5%	1.00
Contralateral breast cancer	1	0.2%	0	0.0	1	0.8%	0.309
Distant recurrence	29	6.7%	19	6.4	10	7.5%	0.680
Local recurrence	24	5.6%	22	7.4	2	1.5%	0.012
IBTR	12	2.8%	18	6.1	1	0.8%	0.010
HER2 patients							
Ipsilateral axillary disease	2	7.1%	1	7.1	1	7.1%	1.00
Contralateral breast cancer	0	0.0%	0	0.0	0	0.0%	
Distant recurrence	2	7.1%	0	0.0	2	14.3%	0.481
Local recurrence	5	17.9%	4	28.6	1	67.1%	0.326
IBTR	4	14.3%	3	21.4	1	7.1%	0.596
TNBC patients							
Ipsilateral axillary disease	5	7.2%	4	9.8	1	3.6%	0.641
Contralateral breast cancer	1	1.4%	1	2.4	0	0.0%	1.00
Distant recurrence	9	13.0%	6	14.6	3	10.7%	0.729
Local recurrence	12	17.4%	11	26.8	1	3.6%	0.021
IBTR	10	14.5%	10	24.4	0	0.0%	0.004

IBTR, ipsilateral breast cancer recurrence.

**Figure 1:**
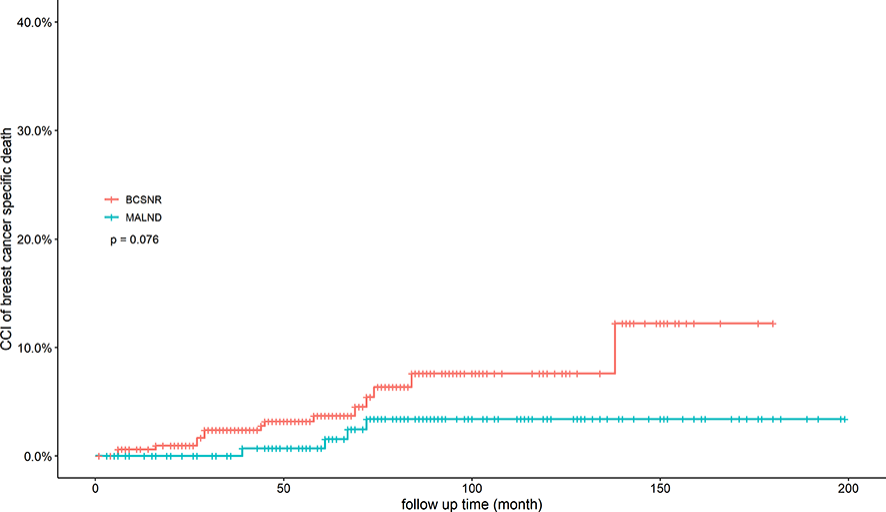
Crude cumulative incidence (CCI) of breast cancer specific death for all patients with BCSNR and MALND.

**Table 3** presents the results of the univariate and multivariable Cox models used to determine the variables influencing distant recurrence. These include surgical method, age group, T stage, histological type, Ki67 expression (low/high), and molecular type. Surgical method had no significant effect on distant recurrence, while T stage and Ki67 expression were both significantly associated with distant recurrence. On Univariate Cox analysis, T stage had significant association with distant recurrence (*p*=0.016). On Univariate Cox analyses and multivariable Cox analyses, Ki67 expression had significant association with distant recurrence (*p*=0.0003, *p*=0.005, conspectively). 5-year crude cumulative incidence of breast cancer-specific death was 3.7% (CI: 1.4%–0.6%) in the BCSNR group and 0.7% (CI: 0%–2.1%) in the MALND group (*p*=0.076) (**Figure 1**).

**Table 3:** Univariate and Multivariable Cox analyses of operation method and other clinical characteristics on distant recurrence.

	Univariate Cox analyses			Multivariable Cox analyses		
	HR	95%CI	*P* value	HR	95%CI	*P* value
Operation method	0.999	0.805-1.240	0.990	0.982	0.770-1.253	0.887
Age group	1.123	0.803-1.570	0.498	1.010	0.706-1.444	0.957
T stage	1.825	1.117-2.982	0.016	1.598	0.950-2.688	0.077
Histological type	0.950	0.714-1.265	0.726	0.910	0.655-1.265	0.575
Ki67(low/high)	3.691	1.799-7.572	0.0003	2.957	1.390-6.292	0.005
Molecular type	1.401	0.963-2.039	0.078	1.182	0.774-1.805	0.439

### Effect of the surgical method on patient survival

**Table 4** presents the rates of breast cancer-specific death, non-breast cancer-specific death, and total death. There was a significant difference between the two groups in total death (*p*=0.040). In the BCSNR group, a total of 57 (15.8%) patients died, compared to 17 (9.4%) patients in the MALND group. However, the difference in breast cancer-specific death was not significant between the two groups (*p*=0.207), with 15 (4.2%) patients in the BCSNR group and 4 (2.2%) in the MALND group dying of breast cancer-specific causes.

**Table 4:** Breast cancer specific death and non-breast cancer specific death according to operation method.

	Total		BCSNR		MALND		
	No	%	No	%	No	%	*P* value
Total patients							
Breast cancer specific death	19	3.5%	15	4.2	4	2.2%	0.207
Non-breast cancer specific death	55	10.2%	42	11.7	13	7.2%	0.089
Total death	74	13.7%	57	15.8	17	9.4%	0.040
Luminal patients							
Breast cancer specific death	11	2.6%	10	3.4	1	0.8%	0.099
Non-breast cancer specific death	45	10.5%	35	11.8	10	7.5%	0.156
Total death	56	13.0%	45	10.5	11	2.6%	0.050
HER2 patients							
Breast cancer specific death	1	3.6%	0	0.0	1	7.1%	0.335
Non-breast cancer specific death	1	3.6%	1	7.1	0	0.0%	0.335
Total death	2	7.1%	1	3.6	1	3.6%	1.00
TNBC patients							
Breast cancer specific death	9	13.0%	5	12.2	4	9.8%	0.504
Non-breast cancer specific death	5	7.2%	2	7.1	3	10.7%	0.958
Total death	14	20.2%	9	13.0	5	7.2%	0.680

**Table 5** presents the results of the univariate and multivariable Cox models used to determine the variables influencing breast cancer-specific mortality, including surgical method, age group, T stage, histological type, Ki67 (low/high), and molecular type. Although surgical method had no significant effect on breast cancer-specific mortality (*p*=0.210, *p*=0.227, respectively in univariate and multivariable Cox model analysis), T stage (*p*=0.001, *p*=0.004, respectively in univariate and multivariable Cox analysis), molecular type (*p*=0.003, *p*=0.042, respectively in univariate and multivariable Cox analysis), and Ki-67 expression (*p*=0.022 in univariate Cox analysis) were all significantly associated with breast cancer-specific death.

**Table 5:** Univariate and Multivariable Cox analyses of operation method and other clinical characteristics on BCSD.

	Univariate Cox analyses			Multivariable Cox analyses		
	HR	95%CI	*P* value	HR	95%CI	*P* value
Operation method	0.790	0.546-1.142	0.210	0.779	0.520-1.168	0.227
Age group	1.488	0.969-2.287	0.070	1.127	0.717-1.772	0.604
T stage	3.271	1.627-6.574	0.001	3.112	1.452-6.672	0.004
Histological type	0.978	0.650-1.472	0.916	0.887	0.555-1.416	0.614
Ki67(low/high)	3.287	1.184-9.127	0.022	1.878	0.616-5.730	0.268
Molecular type	2.081	1.288-3.362	0.003	1.754	1.020-3.018	0.042

BCSD, breast cancer specific death.

**Figure 2:**
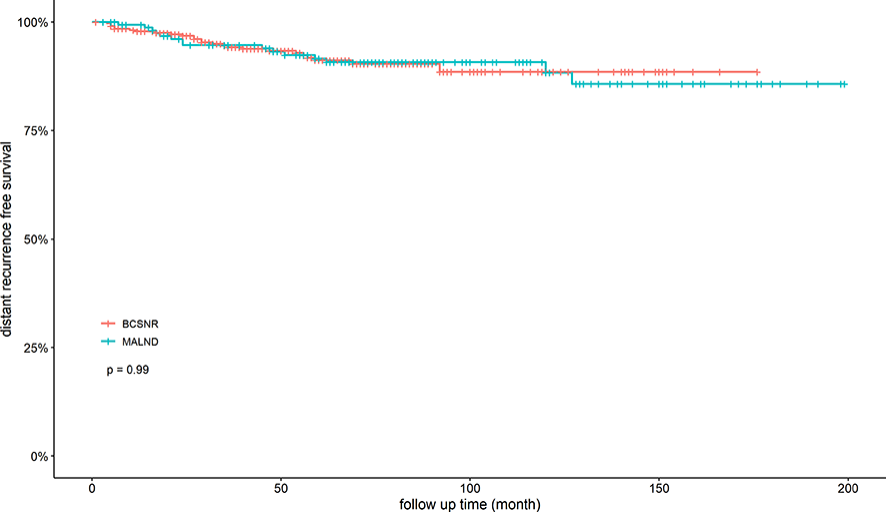
Distant recurrence free survival for all patients with BCSNR and MALND.

**Figure 3:**
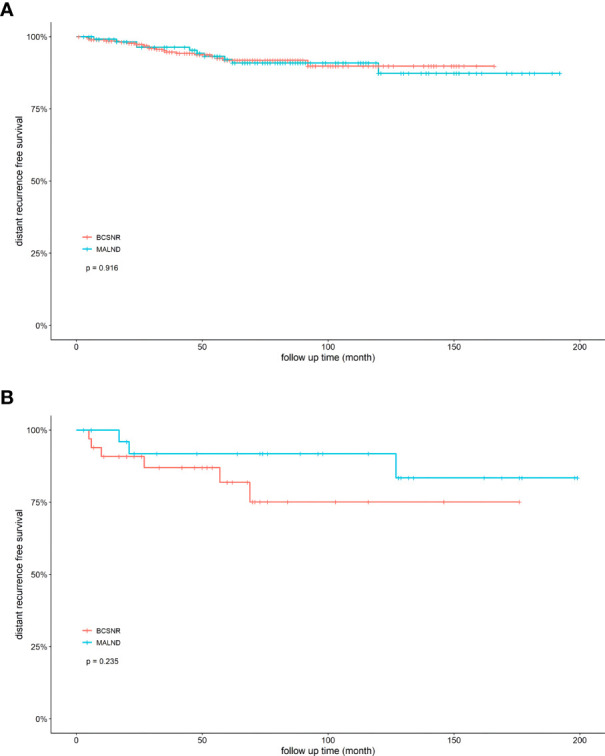
Distant recurrence free survival for elderly breast cancer patients. **(A)** luminal patients with BCSNR and MALND. **(B)** TNBC patients with BCSNR and MALND.

**Figure 4:**
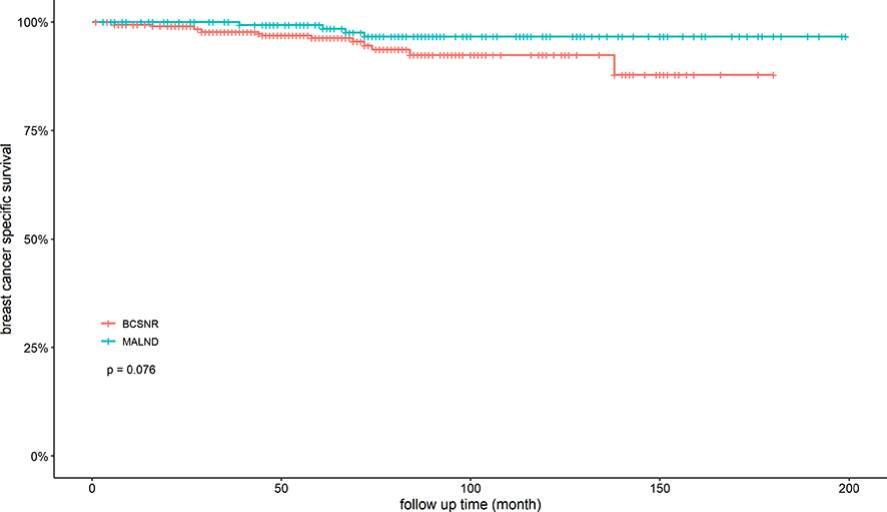
Breast cancer specific survival for all patients with BCSNR and MALND.

We next compared DRFS between the BCSNR and MALND groups (**Figure 2**). The 5-year DRFS was 98.1% in the BCSNR group and 93.2% in the MALND group (*p*=0.990). In luminal type patients, the 5-year DRFS was 91.8% in the BCSNR group and 92.1% in the MALND group (*p*=0.916) (**Figure 3A**), while in TNBC patients, the 5-year DRFS was 81.9% in the BCSNR group and 91.8% in the MALND group (*p*=0.235) (**Figure 3B**). The breast cancer-specific survival rate was 96.3% in the BCSNR group and 99.3% in the MALND group; the difference between the groups was not significant (*p*=0.076) (**Figure 4**).

## Discussion

The prognosis of breast cancer in elderly patients is good, as breast cancer-related mortality declines with advancing age (4). Previous studies have reported that following BCS, radiotherapy can be omitted in some early-stage patients without affecting survival (19). Although radiotherapy technology has made great progress, radiotherapy may cause damage to the heart and lungs (13, 14). It has been proposed that omitting radiotherapy after BCS will not affect the survival of elderly breast cancer patients (20). Likewise, there is evidence to suggest that the survival of elderly breast cancer patients will not be affected if the axillary lymph nodes are not cleaned (11). Therefore, our study sought to determine whether the survival of elderly breast cancer patients with clinically negative axillary lymph nodes is affected by omitting axillary lymph node cleaning and breast and axillary radiotherapy after BCS.

We observed that after a 5-year follow-up, patients over 70 years of age who did not undergo breast and axillary radiotherapy and axillary lymph node dissection had good DRFS and breast cancer-specific survival, and the difference of DRFS and breast cancer-specific survival between those in the BCSNR and MALND groups were not significant. These results were similar to the results from the CALGB9343 study, wherein all the patients had HR-positive and T1 stage disease (21). In our study, 79.3% of patients had HR-positive disease and 96% had a tumor size <5 cm.

Our results indicate that the rates of ipsilateral axillary disease, distant recurrence, and contralateral breast cancer did not differ significantly between the BCSNR and MALND groups. We observed similar findings when comparing patients with luminal type, HER2-enriched type, and TNBC between the groups. Our results are consistent with those of the PRIME II study, in which 90% of the patients had HR-positive tumors and all had tumor sizes <3 cm (12). In our study, the frequencies of patients with HR-negative tumors and patients who had larger tumors were higher.

We did not observe a significant difference in breast cancer-specific deaths between the BCSNR and MALND groups; furthermore, there were no significant differences in breast cancer-specific deaths among patients with luminal type, HER2-enriched, and TNBC between the groups. These results are similar to those reported in the study by Martelli et al. (22) in which 34% of patients received radiotherapy. In our study, no patients received radiotherapy.

We observed a 5-year local recurrence rate of 10.3% in the BCSNR group and 2.2% in the MALND group. Martelli et al. reported that the local recurrence was 12.1% in patients with axillary dissection vs. 7.7% in patients without axillary dissection (22). In their study, T1 patients accounted for 64.1% of the study sample while T2 patients accounted for 29.4%, and the majority (>90%) of patients had HR-positive tumors. In our study, the number of patients with larger tumors was higher (57.3% patients, T1; 28.5% patients, T2), and the number of patients with HR-negative tumors was also higher. The findings of both studies support the conclusion that BCSNR does not affect the survival of elderly patients.

In the present study, the 5-year IBTR rate in the BCSNR group was 8.6% and in the MALND group was 1.1%. Wickberg et al. reported a 5-year IBTR of only 1.2% (23), but it should be noted that in their study, the median tumor size was 1.1 cm, and all patients had HR-positive tumors. The reason for the high IBTR in our study could be that our patients had larger tumor sizes and that some of them had HR-negative tumors. In our study, the IBTR rate was significantly higher in the BCSNR group than in the MALND group. The difference remained significant when the IBTR rate was compared between the TNBC and luminal type patients in each group but not while comparing patients with HER2-enriched type. According to previous studies, elderly breast cancer patients diagnosed with HER2-enriched type or TNBC are more likely to show relapse (24). The discrepancy in our findings could be due to the small size of the HER2-enriched population in our study.

Although the prognosis of elderly patients is good, they cannot completely forego surgical treatment. One study included patients over 70 years of age with clinically negative lymph nodes who had a tumor size <5 cm and high ER expression and randomly divided them into receiving tamoxifen plus mastectomy or tamoxifen alone. Although there was no significant difference between the two groups in breast cancer-related death after 10 years of follow-up, the local recurrence was 43% in the tamoxifen alone group versus 1.9% in the mastectomy plus tamoxifen group (25). In our study, the local recurrence rate in the BCSNR group was 7.6%, indicating that this surgical method is very effective for local control in elderly patients. An analysis of six randomized clinical trials (RCTs) and 31 non-RCTs found that in terms of local control, surgery was a more effective option than endocrine therapy. In patients with a prognosis of more than 5 years, survival seemed to be better with surgery than with endocrine therapy (26), indicating that surgical treatment is very important for elderly breast cancer patients. However, among elderly patients, the 1-year OS rate and relative survival rate are low in patients with postoperative complications, and the risk of postoperative complications increases with the disease stage (6). Therefore, it is beneficial to consider reducing the scope of operations in elderly patients. Previous studies have found that omitting axillary lymph node dissection does not affect the survival of elderly breast cancer patients (11, 22) and that sentinel lymph node biopsies may be omitted in low-risk elderly patients with HR-positive tumors and negative lymph nodes (27). In one study, breast cancer patients over 70 years of age with cT1-2N0 who underwent BCS and non-sentinel lymph node biopsies showed a 5-year OS of 70% and a 5-year breast cancer-related survival rate of 96% (28). The most common cause of death was ischemic heart disease rather than breast cancer (28). In our study, the most common cause of death was non-breast cancer-specific death (10.2%), while breast cancer-specific causes accounted for only 3.5% of deaths. Breast cancer-specific death also did not vary significantly between surgical approaches, with a rate of 4.2% in the BCSNR group compared to 2.2% in the MALND group. These results suggest that omitting radiotherapy and axillary lymph node dissection after BCS does not increase the rate of breast cancer-specific death in elderly breast cancer patients.

In our study, luminal type patients accounted for 79.3% of breast cancer patients, while 5.2% and 12.9% had HER2-enriched type and TNBC, respectively. This is similar to data from previous studies carried out in elderly breast cancer patients that reported a lower number of TNBC patients than that of luminal type patients (10). Breast cancer-specific death was higher in TNBC patients than in luminal type patients (13.0% vs. 2.6%), consistent with the results of previous studies (29). In our study, there was no significant difference in the 5-year DRFS between the BCSNR and MALND groups in patients with either luminal type breast cancer or TNBC. This demonstrates that BCSNR can also be used in early TNBC populations without affecting patient survival.

This study has some limitations. In our study, the total number of HER2-enriched type breast cancer and TNBC patients was small. In the future, we intend to include more non-luminal type patients and carry out prospective studies to further verify the current conclusions. Although this is a retrospective study with a small sample size of HER2-enriched type breast cancer and TNBC patients, to the best of our knowledge, it is the only study on elderly patients aged over 70 years with clinically negative axillary lymph nodes which compared BCSNR and MALND. Furthermore, the baseline characteristics between the BCSNR and MALND groups were more consistent. We also compared the rates of survival, recurrence, and IBTR between the BCSNR and MALND groups, providing a more comprehensive set of results than that could be obtained in a more restricted cohort. In addition, the total number of patients was 541, with a lengthy median follow-up time of 61 months. Over this period, we recorded 74 deaths, 41 relapses, and 40 distant metastases. Our results indicate that for patients over 70 years of age, a 60-month follow-up duration is sufficient to gather conclusive evidence supporting a more conservative therapeutic approach.

In conclusion, BCSNR does not affect the breast cancer-specific survival of elderly breast cancer patients over 70 years of age who have clinically negative lymph nodes. BCSNR does not negatively affect the DRFS of patients compared to MALND, and only local recurrence was significantly different between the two group. Therefore, BCSNR is a safe treatment option for elderly patients with clinically negative axillary lymph nodes.

## Data availability statement

The original contributions presented in the study are included in the article/**Supplementary Material**. Further inquiries can be directed to the corresponding author.

## Ethics statement

The studies involving human participants were reviewed and approved by the IRB of Peking Union Medical College Hospital. The patients/participants provided their written informed consent to participate in this study.

## Author contributions

YinZ wrote the main manuscript text. ZW reviewed the information of patients. YidZ and FM collected and arranged patients’ data. SS, YX conducted statistical analysis. QS supported quality management and directed team. All authors contributed to the article and approved the submitted version.

## Supplementary material

Supplementary Figure 1 Management flow chart of patients.
