# Supplementary figures and images for: Breast-conserving surgery without axillary surgery and radiation versus mastectomy plus axillary dissection in elderly breast cancer patients: A retrospective study
Source: Front Oncol. 2023 Mar 20;13:1126104. doi: 10.3389/fonc.2023.1126104 (PMC10067658; doi:10.3389/fonc.2023.1126104)

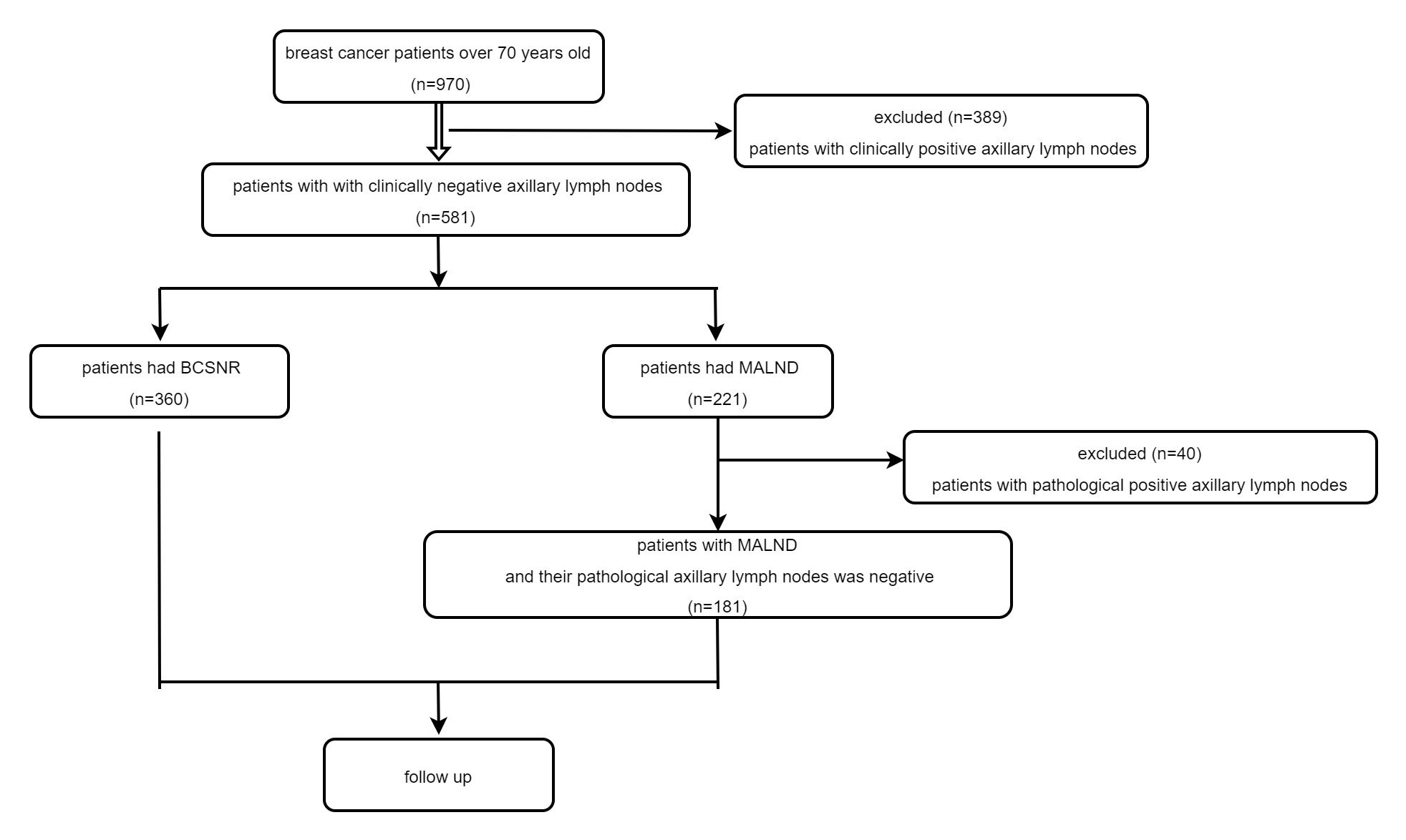

Supplement: Supplementary Figure 1 — Management flow chart of patients. [file Image_1.jpeg]
